# PP30 A Costing Template For The Implementation Of A Public And Patient Advisory Board For Health Research In Dementia

**DOI:** 10.1017/S0266462325102493

**Published:** 2025-12-29

**Authors:** Paola Vasquez, Daniel X Bailey, Paul Prudon

## Abstract

**Introduction:**

Patient and public involvement (PPI) is becoming increasingly embedded in research. While financial constraints are cited as barriers to PPI, there is a lack of understanding of its financial affordability and factors influencing costs. This work aimed to: (i) clarify PPI board costs; (ii) find cost-saving opportunities; and (iii) provide a cost template for others to implement a PPI board.

**Methods:**

A cost analysis was conducted to identify the financial capital and staffing hours required to establish and maintain a PPI board. This analysis involved the following steps: (i) identifying the stages of the project; (ii) outlining the activities associated with each stage; (iii) establishing the time frame for each stage and its activities; (iv) identifying the necessary positions; and (v) calculating the associated costs.

**Results:**

The board’s design, implementation, one-year operation, and evaluation cost AUD150,229 (EUR84,076.41) (43% for design, 21% for implementation, 19% for operation, and 17% for evaluation). Although the distribution of hours allocated per position was consistent with the distribution of costs, the results varied by stage. The highest proportion of hours (and costs) on design (67%) and implementation (46%) were attributed to the head researcher position, whereas the research assistant had the highest proportion of hours for operations (53%). The board members accounted for the lowest proportion of the total hours (5%). No time was allocated to them during the design or evaluation phase, and only 11 and 14 percent were attributed to them for implementation and operation, respectively.

**Conclusions:**

The absence of PPI representatives and junior researchers in the design and evaluation stages led to higher costs and some inconsistencies with the PPI model, which requires public involvement from the outset of any project. A deeper level of PPI from the design of this project would not only make it more financially efficient but also, even more critically, enhance and ensure community engagement.

